# Genome Editing as a Tool for Fruit Ripening Manipulation

**DOI:** 10.3389/fpls.2018.01415

**Published:** 2018-09-25

**Authors:** Carmen Martín-Pizarro, David Posé

**Affiliations:** Laboratorio de Bioquímica y Biotecnología Vegetal, Facultad de Ciencias, Departamento de Biología Molecular y Bioquímica, Instituto de Hortofruticultura Subtropical y Mediterránea, Universidad de Málaga-Consejo Superior de Investigaciones Científicas, Málaga, Spain

**Keywords:** fruit ripening, fruit quality, crops, tomato, genome editing, TALENs, CRISPR/Cas9

## Abstract

Over the last few years, a series of tools for genome editing have been developed, allowing the introduction of precise changes into plant genomes. These have included Zinc-finger nucleases (ZFNs), transcription activator-like effector nucleases (TALENs), and CRISPR/Cas9, which is so far the most successful and commonly used approach for targeted and stable editing of DNA, due to its ease of use and low cost. CRISPR/Cas9 is now being widely used as a new plant breeding technique to improve commercially relevant crop species. Fruit ripening is a complex and genetically controlled developmental process that is essential for acquiring quality attributes of the fruit. Although the number of studies published to date using genome editing tools to molecularly understand or improve fruit ripening is scarce, in this review we discuss these achievements and how genome editing opens tremendous possibilities not only for functional studies of genes involved in fruit ripening, but also to generate non-transgenic plants with an improved fruit quality.

## Introduction

Fruit ripening is a complex and irreversible developmental process that involves numerous metabolic, biochemical, physiological and organoleptic alterations. Among these changes, ripening leads to fruit softening, accumulation of sugars, volatile compounds and pigments, reduction of organic acids, etc., making the fruit more attractive for animal consumption, and therefore, facilitating seed dispersal (Gapper et al., 2013).

Fleshy fruits are classified as climacteric or non-climacteric, depending on whether or not they produce autocatalytic ethylene, respectively. Thus, climacteric fruits such as tomato, apple, avocado, and banana are characterized by an increase in the respiration rate and a burst of ethylene at the onset of ripening (Giovannoni, 2004). In contrast, in non-climacteric fruits, which include strawberry, grape, citrus, and pepper among others, ethylene production remains at low levels and there is no dramatic change in respiration (Symons et al., 2012).

The role of phytohormones and the transcriptional regulation of climacteric and non-climacteric fruit ripening have been extensively reviewed in the last few years (Gapper et al., 2013; Cherian et al., 2014; Karlova et al., 2014; Kumar et al., 2014). In particular, ethylene perception and signaling have been very well characterized, especially in tomato (*Solanum lycopersicum*), which is the most studied model system for fruit ripening (Giovannoni, 2001; Barry and Giovannoni, 2007). In contrast, the regulatory network involved in non-climacteric fruit ripening has been much less studied. Nevertheless, it is known that abscisic acid rather than ethylene is essential in the control of ripening in strawberry (Chai et al., 2011; Jia et al., 2011), the established model for non-climacteric fruits.

Fruit ripening is of major economic importance for agriculture. One of the main challenges for producers is to offer a product at the ripening stage with a good flavor and nutritional value, while also having sufficient shelf life to maintain its quality until it is consumed. This is especially relevant for climacteric fruits highly sensitive to ethylene, and for non-climacteric fruits such as strawberry, which become quickly inedible. Thus, improved ripening and shelf life has been a focus of interest for many scientists in recent decades, using conventional breeding and genetic modification. However, the latter relies on the generation of transgenic plants, which have a very limited commercial use due to the current skepticism of consumers and restrictive government policies. Moreover, transgenic strategies have been based on the modulation of gene expression, which may lead to temporary and/or incomplete knockdown effects, unpredictable off-target effects, and too much background noise (Martin and Caplen, 2007). However, the availability of genome editing tools offers new opportunities to overcome these drawbacks.

## The Emergence of Genome-Editing Technology

In the past decade, new and powerful approaches have emerged enabling the precise editing of a gene of interest. These approaches are based on the use of nucleases that are targeted to a specific sequence to generate a double-strand break (DSB). DSBs trigger two different repair mechanisms: (i) error-prone non-homologous-end-joining (NHEJ) and (ii) homology-directed repair (HDR). While NHEJ repair results in InDel mutations of variable lengths, HDR can be used to introduce specific point mutations or a sequence of interest, through recombination supplying an exogenous donor template. To obtain DSBs for genome editing, four major classes of customizable DNA-binding proteins have been engineered so far: meganucleases (Smith et al., 2006), zinc-finger nucleases (ZFNs) (Urnov et al., 2005), transcription activator-like effector nucleases (TALENs) (Christian et al., 2010), and RNA-guided DNA endonuclease Cas9 (Jinek et al., 2012). Meganucleases, ZFN, and TALEN rely on the binding and recognition of the nucleases to specific sequences of DNA. Therefore, these approaches require complex engineering processes to produce custom nucleases that target the sequence of interest. However, methods based on the bacterial CRISPR (clustered regularly interspaced short palindromic repeats)/Cas system have opened up tremendous possibilities, since the specificity does not lie in the endonuclease, but on a simple and cheap design of a single guide RNA (sgRNA) that is complementary to the target sequence.

Despite the enormous number of reported studies using genome editing technology for gene functional studies in plants and crop improvement (Malzahn et al., 2017), only a small handful of studies, summarized in this review (**Table 1**), have focused on improving or identifying key regulators of fruit ripening as an essential developmental process and an economically relevant trait.

**Table 1:** List of applications of genome editing technologies to study tomato fruit ripening, and CRISPR/Cas9 in various fruit crop species.

Technology	Gene edited in tomato	Character	Reference
TALEN	*PROCERA* (*PRO*)	GA metabolism	Lor et al., 2014
TALEN	*LEAFY COTYLEDON1-LIKE4* (*LIL4*)	Pleiotropic effects	Hilioti et al., 2016
CRISPR/Cas9	*RIPENING-INHIBITOR* (*RIN*)	Fruit ripening	Ito et al., 2015, 2017
CRISPR/Cas9	*PECTATE LYASE* (*PL*)	Fruit firmness	Uluisik et al., 2016
CRISPR/Cas9	*SELF-PRUNING 5G* (*SP5G*) and *SELF-PRUNING* (*SP*)	Photoperiodic response	Soyk et al., 2016
CRISPR/Cas9	LONG-NON CODING RNA (*lncRNA1459*)	Fruit ripening	Li R. et al., 2018
CRISPR/Cas9	*ORGANELLE RECOGNITION MOTIF* (*SlORRM4*)	Mitochondrial function	Yang et al., 2017

**Species**	**Gene edited by CRISPR/Cas9**	**Character**	**References**

Grapevine (*Vitis vinifera*)	*MLO-7*	Pathogen resistant	Malnoy et al., 2016
	*IdnDH*	Tartaric acid biosynthesis	Ren et al., 2016
	*VvPDS*	Carotenoid biosynthesis	Nakajima et al., 2017
Watermelon (*Citrullus lanatus*)	*ClPDS*	Carotenoid biosynthesis	Tian et al., 2016
Cucumber (*Cucumis sativus*)	*eIF4E*	Virus resistance	Chandrasekaran et al., 2016
Banana (*Musa* ×*paradisiaca)*	*PDS*	Carotenoid biosynthesis	Kaur et al., 2018
Kiwifruit (*Actinidia Lindl*)	*AcPDS*	Carotenoid biosynthesis	Wang et al., 2018
Sweet orange (*Citrus sinensis*)	*CsPDS*	Carotenoid biosynthesis	Jia and Wang, 2014
	*CsLOB1*	Pathogen resistant	Peng et al., 2017
Duncan grapefruit (*Citrus* ×*paradisi*)	*CsLOB1*	Pathogen resistant	Jia et al., 2016
	*CsLOB1*	Pathogen resistant	Jia et al., 2017
Apple (genus *Malus*)	*PDS*	Carotenoid biosynthesis	Nishitani et al., 2016
	*DIPM1*, *2* and *4*	Pathogen resistant	Malnoy et al., 2016
Woodland strawberry (*Fragaria vesca*)	*TAA1* and *ARF8*	Auxin biosynthesis and signaling	Zhou et al., 2018
Cultivated strawberry (*Fragaria* ×*ananassa*)	*FaTM6*	Flower development	Martín-Pizarro et al., 2018


## First Genome Editing Approaches in Tomato

In the case of dicot crops, tomato became the ideal candidate for gene editing because of its several advantages, i.e., (i) diploid and high-quality sequenced genome, (ii) ease of transformation, (Van Eck et al., 2006), and (iii) economic importance, being the fourth most important commercial crop in the world. The first reports on genome editing in tomato appeared in 2014 when CRISPR/Cas9 was applied to effectively perform gene functional analysis by stable root transformation, using *Agrobacterium rhizogenes* (Ron et al., 2014). This study was followed by two others, where CRISPR/Cas9 and TALENs were applied to generate mutations in complete plants for the first time, in particular for the *ARGONAUTE7* (*SlAGO7*) and *PROCERA* (*PRO*) genes, respectively (Brooks et al., 2014; Lor et al., 2014). As in most of the pioneer studies of genome editing in any species, both genes had been functionally characterized already, allowing the functional validation of these new approaches. Particularly, *PRO* encodes for a DELLA protein that acts as a negative regulator of gibberellin (GA) signaling (Bassel et al., 2008; Jasinski et al., 2008). Lor et al. (2014) characterized the vegetative stage of the TALEN-induced *pro* mutants, which showed a phenotype consistent with an increased GA response, such as tall and slender plants. Besides their role in plant growth and development, the role of GAs in fruit set (Kumar et al., 2014) and fruit ripening (Dostal and Leopold, 1967) have been widely studied. In fact, a previous report where the spontaneous *pro* mutant was phenotypically characterized, showed that fruit ripening was significantly delayed and that the Brix index value was higher in the mutant (Carrera et al., 2012), consistent with a higher GA response in these plants. Therefore, it would be expected that fruit ripening was also altered in the TALEN-induced *pro* mutants, though Lor et al. (2014) did not characterize the fruit phenotype.

Two years later, the ZFNs gene editing tool was applied for the first time in tomato to mutagenize the *LEAFY COTYLENDON1-LIKE4* (*LIL4*) gene, which encodes for a subunit of a heterotrimeric transcription factor (Hilioti et al., 2014). Mutation in *LIL4* resulted in a pleiotropic phenotype, including fruits with different sizes and shapes, a reduced number of locules, and absence of placenta. Furthermore, fruits with a paler color and slower ripening were obtained (Hilioti et al., 2016), although how *LIL4* regulates this processes is still unknown.

## *RIN* – How CRISPR/Cas9 Converted a Loss-of-Function into a Gain-of-Function Mutation

A large number of studies have been focussed in the role of different transcription factors (TFs) involved in the ripening process. One of the most investigated TFs for ripening is *RIPENING-INHIBITOR* gene (*RIN*), a member of the *SEPALATA* (*SEP*) class of the MADS-box gene family, first discovered half a century ago when a mutation in this locus (*rin*) was found to cause a failure to ripen in tomato (Robinson and Tomes, 1968; Vrebalov et al., 2002). *RIN* is induced early during ripening, and regulates ethylene-dependent and ethylene-independent pathways that promote ripening (Karlova et al., 2014). The effect on ripening of the *rin* mutation has been commercially exploited as hybrid cultivars (*RIN/rin*) with an extended shelf life (Kitagawa et al., 2005). Due to the importance and clear phenotype of the *rin* mutation, the *RIN* locus has been recently targeted using CRISPR/Cas9 to validate the functionality and inheritance of mutations mediated by this approach in tomato. Ito et al. (2015) designed three CRISPR/Cas9 constructs to mutagenize three different regions within the *RIN* locus. As expected, CRISPR/Cas9-mediated novel mutations at the *RIN* locus resulted in an inhibition of fruit ripening at the T0 generation (Ito et al., 2015). However, and contrary to the *rin* mutant, these CRISPR mutants partially initiated the ripening process, and this was interpreted as the result of the presence of wild-type RIN in the T0 generation. Previously, Vrebalov et al. (2002) generated knockdown *RIN* plants using RNA interference, resulting in a fruit ripening that was only partially suppressed, in contrast to the green *rin* mutant phenotype, and interpreted as due to residual expression of *RIN.* Thus, RIN has been considered so far to function as an essential regulator of ripening, and the models have always been based on the idea that *rin* was a loss-of-function mutation. However, a recent paper has overturned this model (Ito et al., 2017). Firstly, unlike the *rin* mutant, homozygous CRISPR/Cas9-mediated knockout *rin* mutants (*RIN-*KO) did not fail to ripen, reaching a pale red color. Moreover, a molecular and physiological characterization of these lines showed that most ripening-related parameters were less affected than in the *rin* mutant. These results suggested that, contrary to what has been considered so far, ripening can be initiated independently of RIN. Furthermore, they also suggested that the *rin* mutant protein may have gained a new function, as a partial dominant negative mutation that blocks the initiation of ripening. This hypothesis was supported by the fact that *rin* mutant allele encodes for a in-frame fusion of *RIN* and *Macrocalyx* coding sequences (Vrebalov et al., 2002), the latter containing a putative repression motif that might convert *rin* into a transcriptional repressor. This repressor activity was actually confirmed *in vitro* (Ito et al., 2017). Consistent with this hypothesis, use of CRISPR/Cas9 to generate additional mutations in the *rin* mutant allele (*rinΔN*) resulted in fruits that recovered the initiation of ripening, showing a similar phenotype to that of *RIN-*KO lines. Furthermore, a molecular and physiological characterization of *rinΔN* lines showed a partial recovery of most of the ripening markers. Thus, this study proposes that the *rin* mutant protein would impair the DNA-binding and activation of ripening-related genes by other master regulators such as NONRIPENING (NOR), COLORLESS NONRIPENING (CNR) (Giovannoni, 2004; Manning et al., 2006), or other SEP homologs.

In conclusion, the use of a gene editing approach such as CRISPR/Cas9 has allowed generating alternative knockout alleles, which have changed our current model of fruit ripening, with RIN being necessary to initiate this process, and *rin* being a loss-of-function mutation. This implies that many results obtained in the past should be reconsidered, and that further experiments should be carried out now that we are closer to defining the actual mechanism.

## Targeting Fruit Texture

While *RIN/rin* hybrid plants are widely used by tomato breeders, the incomplete ripening of these hybrid fruits leads frequently to a poor flavor and a reduced nutritional value. Hence, to modify texture characteristics for an improved shelf life, without reducing tomato organoleptic and nutritional quality, has been a challenge for researchers and breeders for many years. Fruit softening depends on cell-wall modifying proteins such as polygalacturonase, pectin methylesterase, endo-β-(1,4)-glucanase, β-galactosidase, and expansin. A number of studies have characterized the effect of silencing the expression of genes encoding these proteins in strawberry (Posé and García-Gago, 2011), and tomato (Sheehy et al., 1988; Smith et al., 1990, 1988; Tieman et al., 1992; Hall et al., 1993; Tieman and Handa, 1994; Brummell et al., 1999; Smith, 2002). Silencing of the polygalacturonase gene had no apparent effect on tomato fruit softening (Sheehy et al., 1988; Smith et al., 1990, 1988), but it did affect the firmness of strawberry fruits, which even showed a slightly higher °Brix (Quesada et al., 2009). For the rest of the genes, silencing of their expression has had only very small or no detectable effects on both tomato or strawberry fruit ripening (Tieman et al., 1992; Hall et al., 1993; Tieman and Handa, 1994; Brummell et al., 1999; Smith, 2002). However, silencing another cell-wall related protein, the pectate lyase (PL), has been successfully applied for the modulation of fruit firmness in both species. *PL* silencing increased fruit firmness without changes in color, size, total soluble solids, or metabolites influencing taste and aroma in both strawberry (Jiménez-Bermúdez et al., 2002), and tomato (Uluisik et al., 2016). Particularly in tomato, preliminary analysis of CRISPR/Cas9-induced *pl* mutants has shown an effect on fruit firmness without altering color and soluble solids content (Uluisik et al., 2016). A further characterization would be necessary to confirm that these CRISPR/Cas9 mutant lines maintain other important agronomical characteristics such as aroma, flavor, yield, color, and resistance to pathogens, all required traits for a successful introduction to the market.

## Targeting Photoperiodic Response

An appropriate timing of flowering is not only essential for plant reproductive success but also to optimize yield in agriculture. In a search for the genetic component controlling the different day-length sensitivities regulating flowering in tomato, Soyk and colleagues identified *SELF PRUNING 5G* (*SP5G*) as the responsible gene (Soyk et al., 2016). *SP5G* is a *FLOWERING LOCUS T-*like gene that works as a floral repressor controlling flowering under long-day conditions (Cao et al., 2015). In this study, the authors generated CRISPR/Cas9-mediated mutations for this gene, obtaining plants that flowered earlier under long-day conditions. Another gene, *SELF-PRUNING* (*SP*), is an ortholog of Arabidopsis *TERMINAL FLOWER 1* (*TFL1*) and encodes for another flowering repressor in tomato (Pnueli et al., 1998). The *sp* mutation revolutionized tomato cultivation since it leads to determinate plants with a synchronized burst of flowering and fruit ripening. In order to obtain faster-flowering and determinate growth plants, Soyk and collaborators used CRISPR/Cas9 to generate double *sp5g sp* mutants, which showed an earlier flowering burst and an earlier fruit ripening than that of the *sp* single mutant and the wild-type (Soyk et al., 2016). However, the earlier tomato ripening in the double *sp5g sp* mutant might be caused simply by the earlier flowering time phenotype of these plants. Therefore, further studies on fruit ripening dynamics need to be performed to clarify whether SP5G actually modulates actively this process.

## Targeting Post-Transcriptional Regulation

There are several previous studies demonstrating the importance of post-transcriptional regulation by non-coding RNAs in the control of fruit ripening (Moxon et al., 2008; Zhu et al., 2015; Wang et al., 2016). To investigate further, CRISPR/Cas9 has been employed in two studies to identify and characterize post-transcriptional regulators of tomato fruit ripening.

In plants, long non-coding RNAs (lncRNAs) are important regulators of gene expression, as they interact with DNA, RNA and proteins (Zhu and Wang, 2012). Interestingly, two lncRNAs, lncRNA1459 and lncRNA1840, have been recently associated with tomato fruit ripening (Zhu et al., 2015). To investigate further the role of lncRNA1459 in fruit ripening, this gene was stably knocked-out by Li X. et al. (2018) using CRISPR/Cas9, obtaining CR-*lncRNA1459* mutant lines with a delayed fruit ripening. A molecular characterization of this mutant showed that key ripening-related genes involved in lycopene and ethylene biosynthesis, and in signal transduction were down-regulated. Consistently, CR-*lncRNA1459* mutant fruits showed a reduction in lycopene accumulation and an inhibition of ethylene production (Li R. et al., 2018). However, the mechanism and target genes of lncRNA1459 in its regulation of fruit ripening still need clarification.

Another post-transcriptional regulation involved in ripening that has been recently explored is RNA editing. In flowering plants, RNA editing by cytidine-to-uridine (C-to-U) conversion is a widespread process that occurs only in plastids and mitochondrial transcripts and plays an important role in developmental processes such as organelle biogenesis, adaptation to environmental changes and signal transduction (Ichinose and Sugita, 2017). In a recent report, Yang et al. (2017) aimed to identify RNA editing factors that might play an essential role in the regulation of tomato fruit ripening. A virus-induced gene silencing (VIGS) assay was performed, targeting 11 RNA editing factor genes putatively related to ripening, positively identifying *SlORRM4*, which is located in mitochondria (Yang et al., 2017). Consistently, CRISPR/Cas9-mediated stable *slorrm4* mutants resulted in a delay of ripening, and in a diminution of the respiratory rates and ethylene production compared with the wild-type. Further molecular characterization showed that *slorrm4* mutation results in a down-regulation of genes associated with the Krebs cycle and mitochondrial function, and a decrease in the level of proteins essential for the mitochondrial respiratory chain, supporting the essential role of mitochondria in the regulation of ripening. However, the specific mechanisms linking RNA-editing in mitochondria with ripening requires further investigation.

## Future Perspectives

Besides ripening, other interesting agronomic traits have been modulated recently in tomato using CRISPR/Cas9, such as parthenocarpy (Klap et al., 2017; Ueta et al., 2017), lycopene content (Li X. et al., 2018), and fruit size, inflorescence branching and plant architecture (Rodríguez-Leal et al., 2017). Especially relevant is the work of Rodríguez-Leal et al. (2017), in which, instead of editing CDS loci, they targeted *cis-*regulatory elements (CREs) in promoters, obtaining quantitatively different phenotypes (Rodríguez-Leal et al., 2017). CRE mutations are widespread in nature and have notably contributed to crop domestication through the alteration of gene expression levels (Meyer and Purugganan, 2013). Thus, targeting CREs with genome-editing technologies offers the possibility to fine-tune gene expression without the common pleiotropic effects observed in complete loss-of-function mutants, opening the door to enhance variability for important agronomic and quality traits. However, a lack of precise knowledge about functional motifs in CREs hampers the current application of this approach.

In addition to tomato, the CRISPR/Cas9 gene-editing strategy has been successfully applied in several fruit crop species to date, including examples of climacteric ripening species, such as apple (Malnoy et al., 2016; Nishitani et al., 2016), banana (Kaur et al., 2018) and kiwifruit (Wang et al., 2018), and non-climacteric ripening species, such as sweet orange (Jia and Wang, 2014; Peng et al., 2017), Duncan grapefruit (Jia et al., 2016, 2017), grapevine (Malnoy et al., 2016; Ren et al., 2016; Nakajima et al., 2017), watermelon (Tian et al., 2016), cucumber (Chandrasekaran et al., 2016), and the woodland (Zhou et al., 2018) and cultivated strawberries (Martín-Pizarro et al., 2018; **Table 1**). Most of these studies have targeted either the *Phytoene Desaturase* gene (*PDS*), or genes to improve pathogen resistance. However, they have opened up the possibility of using CRISPR/Cas9 technology to study or improve fruit ripening in these crops. Among them, strawberry is a species of particular interest because the fast softening of the berries is the main cause of its short shelf life and the source of commercial losses (Perkins-Veazie, 1995). Hence, the successful application of gene editing using the CRISPR/Cas9 approach may provide effective solutions for these postharvest issues.

It is important to highlight that all these studies are based on the generation of new random mutations mediated by the NHEJ mechanism. However, homologous recombination-based gene targeting (GT) allows a more precise genome editing. GT has already been successfully achieved in several crops, including tomato (Čermák et al., 2015; Filler Hayut et al., 2017). In a recent study, GT has been used to extend tomato shelf life by the incorporation of *alcobaca* (*alc*) (Yu et al., 2017), an allelic mutation of *NOR* with a lower impact on fruit quality than *nor* and *rin* mutations (Casals et al., 2011), into a wild-type genotype. Despite these successful studies, GT is still a very challenging approach due to its low efficiency. Thus, the optimization of GT in higher plants in general, and in crop species in particular, would provide of a much wider spectrum of possibilities for breeding, allowing the introgression of genes of interest into elite breeding germplasm.

**FIGURE 1:**
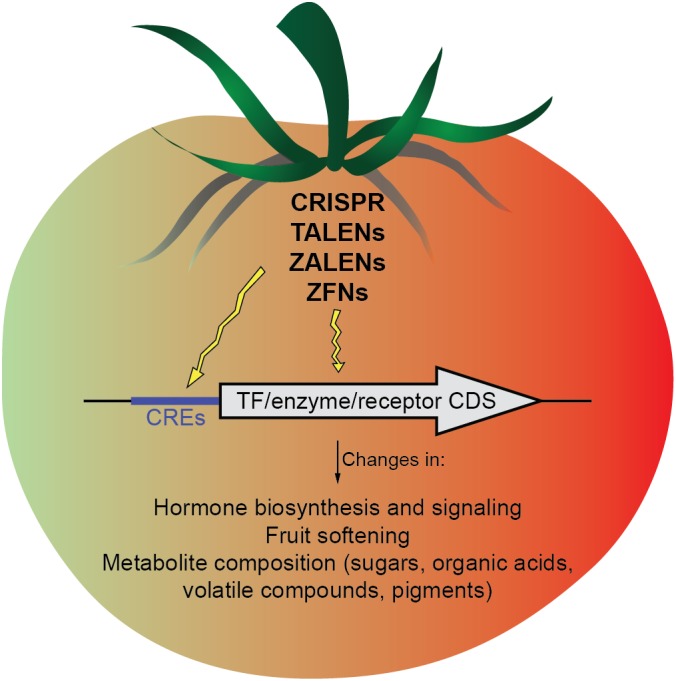
Scheme of the possible targets (*cis-*regulatory elements and gene coding sequences) that might be modified by genome-editing approaches to modulate different aspects of fruit ripening.

In conclusion, genome editing strategies, especially CRISPR/Cas9 are becoming rapidly more efficient and precise. Their application to the coding sequences of TFs, hormones or metabolites biosynthetic enzymes, and hormone receptors, or, alternatively to CREs of these genes may allow a more precise fruit ripening modulation (**Figure 1**). Importantly, genome editing tools have the possibility of removing transgenes through self- or backcrossing, an important advantage compared to traditional approaches for genetic modification. Moreover, preassembled Cas9 protein-gRNA ribonucleoproteins (RNPs) remove the likelihood of inserting recombinant DNA into the host genome. This particular approach has been demonstrated in the protoplasts of several plant species (Woo et al., 2015; Malnoy et al., 2016; Svitashev et al., 2016; Liang et al., 2017) as a strategy that could be potentially operated outside existing GMO regulatory criteria and gain acceptance from consumers. However, the recent decision of the Court of Justice of the European Union is a major setback to innovation in EU agriculture, considering the “process” instead of the “product” and putting crops created using gene-editing techniques under GMO regulations (Directive 2001/18/EC). Hopefully, these regulatory decisions will be reconsidered in the future, as there are unquestionable advantages of gene editing to address the challenge of ensuring sufficient food supply for an increasing global population in the current changing climatic conditions.

## Author Contributions

All authors conceived and wrote the manuscript.

## Conflict of Interest Statement

The authors declare that the research was conducted in the absence of any commercial or financial relationships that could be construed as a potential conflict of interest.
